# Digital Manufacturing of Calcium Phosphate-Based Dental Materials: From Design to Clinical Use

**DOI:** 10.3390/ma19040757

**Published:** 2026-02-15

**Authors:** Valentin Lamasanu, Ionut Luchian, Dragos Ioan Virvescu, Teona Tudorici, Zinovia Surlari, Dana Gabriela Budala, Florinel Cosmin Bida, Oana Maria Butnaru, Mihaela Scurtu, Andrei Georgescu, Nicoleta Ioanid, Monica Silvia Tatarciuc

**Affiliations:** Grigore T. Popa University of Medicine and Pharmacy, 700115 Iasi, Romania

**Keywords:** calcium phosphate materials, dental biomaterials, digital manufacturing, additive manufacturing, material processing, scaffold

## Abstract

Calcium phosphate-based materials are widely used in dental applications due to their bioactivity and compositional similarity to mineralized tissues. This narrative review synthesizes the peer-reviewed literature published between 2014 and 2024 identified using Scopus, Web of Science, and PubMed, with a specific focus on digitally manufactured calcium phosphate-based dental materials. This review integrates evidence on calcium phosphate phase chemistry and advanced material systems, material configurations compatible with digital workflows and digital manufacturing routes. Emphasis is placed on how fabrication parameters and post-processing—particularly thermal treatment and densification—affect phase composition, microstructure/porosity, mechanical performance, and biological behavior. The current limitations reported in the literature are addressed, including material brittleness, dimensional instability during sintering, heterogeneity of fabrication protocols, and the lack of standardized evaluation methods. The restricted availability of long-term and clinically relevant evidence is also discussed in relation to translational applications. Future research should prioritize well-designed clinical studies to validate the use of digitally manufactured calcium phosphate-based materials in guided bone regeneration, socket preservation, and implant-related procedures.

## 1. Introduction

The continuous evolution of digital technologies has profoundly transformed contemporary dental materials science, enabling unprecedented levels of precision, customization, and biological performance.

To enhance their efficacy and influence on patients’ everyday lives, biomedical device advancements are essential. Because of their similarities to bone tissue, which is mostly constituted of carbonated hydroxyapatite, calcium phosphate-based materials have seen extensive usage and development in medical and dental domains [1,2].

In parallel, advances in digital manufacturing, including additive manufacturing and computer-aided subtractive workflows, have reshaped the development, production, and clinical implementation of dental biomaterials [3]. Digital processes allow for the regulation of the geometry, internal architecture, and microstructural characteristics of calcium phosphate-based systems very precisely [4]. This is important for improving their biological and mechanical behavior. These features are especially useful for applications that need custom porosity, controlled degradation rates, and adaptability to each patient [5,6].

Despite growing interest, there is a lack of comprehensive study on computer-made calcium phosphate materials. Many studies neglect important aspects of the problem, such as the biological response, processing factors, or material chemistry. Repeatability, standardization, and long-term effectiveness in oral contexts are particularly problematic areas where there are discrepancies between experimental development and its clinical application [7]. It is essential to conduct a comprehensive synthesis that incorporates material characteristics, digital manufacturing processes, biological efficiency, and therapeutic applicability.

While several reviews have addressed calcium phosphate-based materials in dentistry, most existing studies focus primarily on conventional fabrication routes, surface coatings, or isolated material properties, often without considering the implications of digitally assisted manufacturing workflows. Other reviews emphasize additive manufacturing technologies in dentistry more broadly, but provide limited discussion on calcium phosphate-specific material behavior, phase stability, or processing-induced microstructural changes. As a result, the interdependence between digital fabrication strategies, material-level outcomes, and clinically relevant performance remains insufficiently synthesized. Given the rapid advancement of digital manufacturing technologies and their increasing adoption in regenerative dentistry, a focused and integrative analysis of digitally manufactured calcium phosphate-based dental materials is warranted at this time.

This narrative review aims to provide an overview of calcium phosphate-based dental materials within the framework of digital manufacturing, tracing their development from design and processing considerations to biological behavior and clinical use. By analyzing current evidence, highlighting translational challenges, and identifying future research directions, this work seeks to support the rational development of digitally fabricated calcium phosphate materials and their effective implementation in modern dental practice.

**Figure 1:**
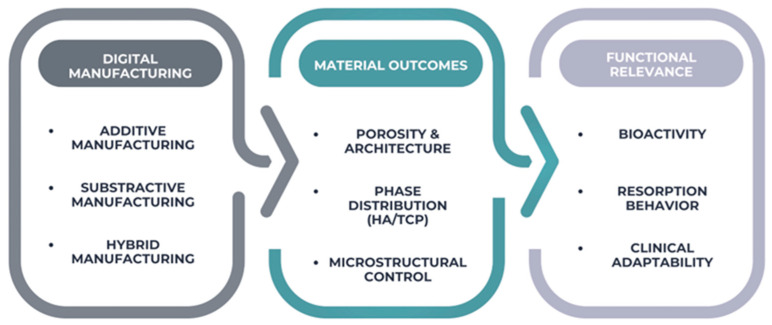
Overview: Digital manufacturing, material outcomes and functional relevance.

The interdependence between digital manufacturing strategies, material-level outcomes, and clinical functionality is schematically illustrated in Figure 1, highlighting the multiscale relationship between processing, structure, and biological performance.

## 2. Literature Review

This narrative review encompasses the most up-to-date information on digitally manufactured calcium phosphate-based dental materials. The literature search was conducted using the Scopus, Web of Science, and PubMed databases. Search terms included combinations of keywords such as “calcium phosphate”, “hydroxyapatite”, “tricalcium phosphate”, “digital manufacturing”, “additive manufacturing”, “3D printing”, “CAD/CAM”, “dental materials”, “bone regeneration”, and “dental scaffolds”.

The search was limited to peer-reviewed articles published in English between 2014 and 2024. Studies were considered eligible if they addressed calcium phosphate-based materials in the context of digital or digitally assisted manufacturing, material processing, biological performance, or dental clinical applications. Exclusion criteria included studies focusing exclusively on non-dental applications, metallic or polymeric systems without calcium phosphate components, or conventional fabrication routes without digital involvement.

An initial screening of titles and abstracts was performed to assess relevance, followed by full-text evaluation of selected articles. The literature search was limited to studies published between 2014 and 2024 to allow for a structured and critical synthesis of available evidence. Publications appearing after this period were not systematically included, as emerging data may require further validation and will be addressed in future updates of the field. Due to the narrative nature of this review, no formal quality scoring or quantitative meta-analysis was performed.

Emphasis was placed on studies published in English and on contributions that provided relevant experimental, translational, or clinically oriented insights. Seminal articles and recent high-impact reviews were also examined to ensure appropriate contextualization of technological developments and emerging trends.

This narrative approach allowed for the integration of diverse study designs, including in vitro investigations, preclinical studies, and clinically oriented reports, facilitating a comprehensive synthesis of current knowledge.

In addition to peer-reviewed journal publications, limited and carefully selected sources of grey literature were consulted to enhance contextual understanding of rapidly evolving digital manufacturing technologies involving calcium phosphate-based dental materials. These sources, such as academic theses, conference proceedings, and technical reports, were used solely to complement the peer-reviewed evidence by providing background on emerging fabrication approaches, technological constraints, and translational considerations. Grey literature was not employed as a primary source of evidence for clinical effectiveness or biological outcomes, and its inclusion was intentionally restricted to avoid methodological bias while preserving the narrative and exploratory nature of this review.

### 2.1. Calcium Phosphate-Based Materials

Because of their structural and chemical similarity to the mineral component of hard tissues, calcium phosphate-based materials have been thoroughly researched for use in dentistry and maxillofacial surgery [8,9]. Applications in oral contexts where biological integration is necessary are ideal for their biocompatibility, bioactivity, and ability to support hard tissue regeneration, which are all based on their compositional closeness to biological apatite [10].

**Figure 2:**
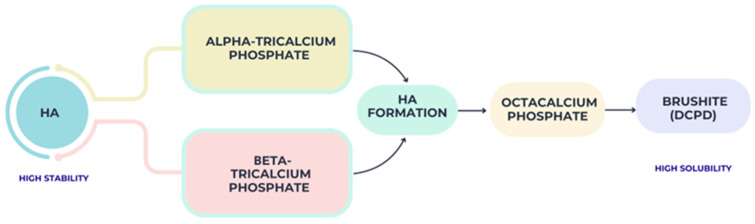
Calcium phosphate phases and transformation pathways.

There are multiple compositional phases in calcium phosphate materials, and each of these phases has its own unique set of physicochemical and biological properties. The most researched type is hydroxyapatite because of its excellent osteoconductive potential and good chemical stability. However, its limited resorption rate may restrict its effectiveness in clinical scenarios where complete material replacement by newly formed bone is desired [11]. The main calcium phosphate phases and their transformation pathways toward biological apatite are schematically summarized in Figure 2.

On the other hand, α- and β-tricalcium phosphate, which are tricalcium phosphate phases, show increased solubility and degradation rates that are faster. One of the more intriguing forms for biomedical uses, especially in bone regeneration, is α-Tricalcium phosphate (α-TCP) [12]. Phases with a Ca_3_(PO4)_2_ composition and a Ca/P molar ratio of 1.5 are chemically referred to as TCP (tricalcium phosphate) in its most literal sense. α-TCP stands out as the TCP phase that occurs at high temperatures. It is usually made by heating β-TCP to temperatures over 1125 °C, then quickly cooling it to avoid its reversal into the more stable β-phase at ambient temperature [13]. The chemical content of α-TCP and β-TCP is identical, but there are notable variations in their crystal structure and, most importantly, their solubility. As a guide for the formation of bone tissue, α-TCP can react and change into hydroxyapatite under normal physiological conditions [14].

Due to its osteoconductive and, in certain instances, osteoinductive qualities, β-tricalcium phosphate (β-TCP) has been one of the most researched and used phases in the realm of biomaterials for bone regeneration. β-TCP is ideal for uses that necessitate slow resorption over time due to its solubility, which falls somewhere in the middle of hydroxyapatite and α-TCP [15]. The inability to immediately precipitate β-TCP from water is a characteristic of this compound that is a result of its chemical and physical stability requirements. The thermal decomposition of carbonated apatite (CDHA) or solid-state reactions involving acidic calcium phosphates and calcium oxide or calcium carbonate at temperatures higher than 800 °C are two other methods for its preparation [16].

Gradual material resorption and contemporaneous bone regeneration are made possible by these features, which allow for more dynamic contact with the surrounding biological environment. It is important to carefully evaluate the clinical indications of tricalcium phosphate due to its lower mechanical stability compared to hydroxyapatite, despite its improved resorbability, which is beneficial in regenerative operations [17].

The primary reasons for studying other calcium phosphate phases including octacalcium phosphate and dicalcium phosphate dihydrate have been their potential to play a role in biological apatite production and their involvement in early-stage mineralization [18]. Despite the positive bioactivity, these phases are not suitable for use as dental materials on their own due to their fast disintegration and low structural integrity [19].

**Figure 3:**
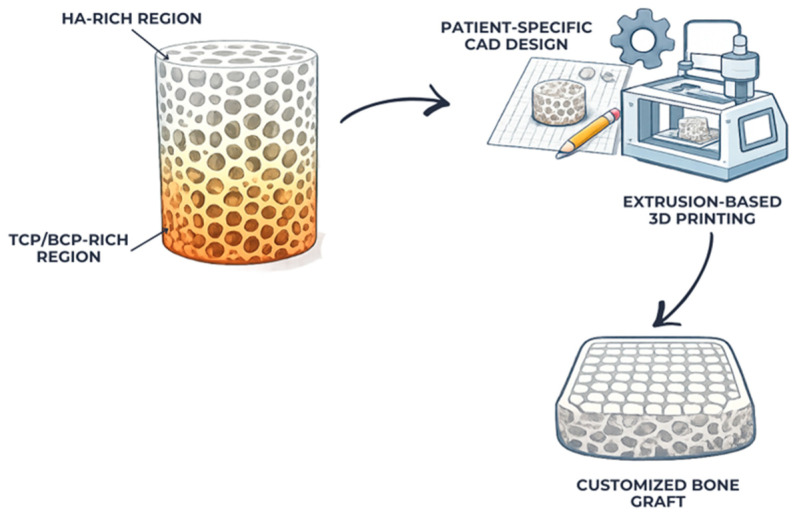
Digital workflow for multiphasic calcium phosphate scaffolds.

Many biphasic and multiphasic calcium phosphate systems have been created to overcome the drawbacks of individual phases. These systems usually incorporate hydroxyapatite and more soluble calcium phosphate phases [20]. Composite systems that allow for phase ratio adjustment provide more leeway for indication-driven and patient-specific applications by allowing for fine-tuning of mechanical stability, degradation rate, and biological response [21]. Precise control over the formulation and design of digitally generated structures makes this kind of compositional tailoring very useful for optimizing clinical performance [22]. The integration of compositional tailoring with digitally assisted design and fabrication enables the development of multiphasic, patient-specific calcium phosphate scaffolds, as conceptually illustrated in Figure 3:

While tricalcium phosphate-containing formulations may be better suited for regenerative settings that benefit from a higher resorption rate and progressive bone replacement, hydroxyapatite-based materials are typically considered when prolonged structural stability and volume preservation are important from a clinical standpoint [23].

From a clinical perspective, the selection of calcium phosphate phases must be guided by the balance between resorption behavior and the need for temporary or prolonged structural support. α-Tricalcium phosphate (α-TCP), owing to its higher solubility and rapid hydrolysis toward apatite, may outperform β-TCP in regenerative scenarios where accelerated material replacement by newly formed bone is desirable, such as early-stage defect filling or applications requiring rapid remodeling. However, this increased reactivity may also limit its suitability in situations requiring prolonged volumetric stability.

In contrast, β-tricalcium phosphate (β-TCP) exhibits a more moderate resorption profile, making it clinically advantageous in indications where gradual degradation and sustained osteoconductive support are required. Nevertheless, its lower mechanical stability compared to hydroxyapatite restricts its use in defects exposed to higher functional loading.

Hydroxyapatite-based materials provide superior chemical stability and volume preservation, which can be beneficial in maintaining space in regenerative procedures. However, their limited resorption may represent a clinical drawback in cases where complete material replacement by native bone is desired, potentially leading to long-term persistence of residual material. To provide a concise overview of advanced and modified calcium phosphate-based dental materials, their key processing-related findings, functional relevance, and associated limitations in the context of digitally assisted fabrication, these aspects are summarized in Table 1.

**Figure 4:**
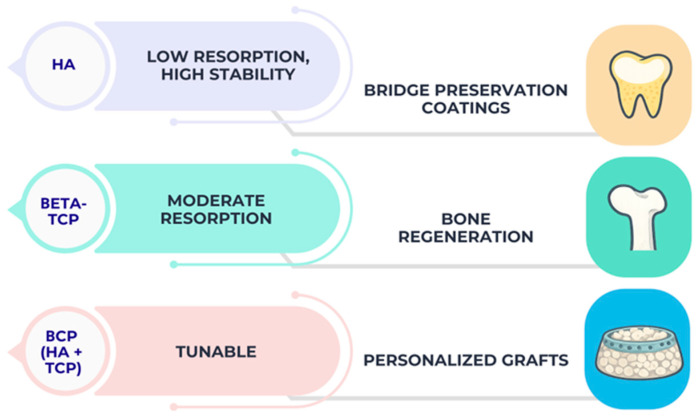
Phase-dependent behavior and dental indications.

A comparative overview of the relationship between calcium phosphate phase composition, resorption behavior and typical dental application is presented in Figure 4.

In addition to compositional phase selection, the performance of calcium phosphate-based dental materials is strongly influenced by intrinsic material characteristics such as crystallinity, particle morphology, Ca/P ratio, and surface chemistry [24]. Variations in crystallinity and crystal sizes can significantly affect dissolution behavior, ion release, and subsequent biological responses, including protein adsorption and cell attachment. Materials with lower crystallinity generally exhibit enhanced bio resorption, whereas highly crystalline phases tend to provide superior chemical stability and prolonged structural integrity [25].

Another important factor controlling the performance of dental calcium phosphate materials is their porosity. By impacting mechanical strength and promoting vascularization, nutritional transport, and cellular migration, macro- and microporosity play a role in biological integration [26]. Finding the balance between mechanical stability and porosity is still a major obstacle, especially for uses that need for volumetric stability in the mouth [27].

Calcium phosphate materials’ interactions with their environments are further modulated by surface characteristics. Studies have demonstrated that chemical functional groups, surface roughness, and wettability all have an impact on the adhesion, proliferation, and differentiation of osteoblasts [28,29]. The use of surface-engineered calcium phosphate materials to improve early-stage biological responses without changing bulk composition is therefore being more and more investigated [30].

### 2.2. Advanced and Modified Calcium Phosphate-Based Dental Materials

Beyond conventional stoichiometric calcium phosphate phases, a wide range of advanced and modified calcium phosphate-based materials have been developed to overcome the intrinsic limitations of single-phase systems and to enhance biological, mechanical, and functional performance in dental applications [31]. These material variants are particularly relevant in the context of modern regenerative dentistry and digitally assisted fabrication strategies.

#### 2.2.1. Ion-Substituted and Doped Calcium Phosphates

One method that has been studied extensively for modifying the physicochemical and biological properties of calcium phosphate compounds is ion substitution [32]. The complex composition of natural bone mineral can be more precisely mimicked by doped calcium phosphates, which include partially substituting calcium or phosphate ions inside the crystal lattice [33]. Silver, strontium, zinc, silicon, and magnesium are common substitutes; they all have different functional consequences [34]. Strontium inclusion, for example, may encourage bone formation and decrease resorption, whereas magnesium and silicon substitutions have been linked to better bioactivity and an enhanced osteogenic response [35]. Doping with zinc and silver has also been studied for its possible antimicrobial effects. By modifying these systems with ions, we can control surface reactivity, cellular interactions, and resorption kinetics precisely without changing the calcium phosphate structure [36].

#### 2.2.2. Composite Calcium Phosphate Systems

Composite materials that combine calcium phosphates with organic or inorganic phases are becoming more popular as a solution to the inherent fragility and low mechanical strength of calcium phosphate ceramics [37]. Improved toughness, flexibility, and processability can be achieved with polymer calcium phosphate composites that incorporate biodegradable polymers such polylactic acid, polycaprolactone, or natural biopolymers. The goal of inorganic composites, such as calcium phosphate-bioglass systems, is to improve mechanical stability, ion release profiles, and osteoconductivity simultaneously [38]. For digitally made structures, where printability, structural integrity, and controlled degradation need to be matched for clinical use, these composite compositions are very beneficial [39].

#### 2.2.3. Amorphous and Metastable Calcium Phosphate Phases

Another group of materials based on calcium phosphate that serve different purposes are amorphous calcium phosphate and other metastable phases. Amorphous calcium phosphate is ideal for uses involving remineralization and early-stage mineral deposition due to its high solubility and quick ion release, in contrast to crystalline phases [40]. Amorphous calcium phosphate is not always the best choice for load-bearing or volumetric applications due to its unstable structure. However, it can be used as a building block for more stable apatite structures and even added to composites or surface-modified systems to make them more biologically responsive [41].

#### 2.2.4. Functional Forms and Material Configurations

From a practical and translational perspective, calcium phosphate-based materials are available in various functional forms, including granules, cements, pastes, and injectable formulations. More recently, tailored inks, slurries, and feedstocks have been developed to support digitally assisted manufacturing processes [42,43]. These material configurations influence handling properties, setting behavior, and suitability for specific clinical indications, while also determining compatibility with additive or hybrid fabrication techniques. The diversity of available forms underscores the adaptability of calcium phosphate materials across both conventional and digitally driven dental workflows [44].

Despite their promising functional advantages, advanced and modified calcium phosphate-based materials are associated with several potential drawbacks that must be carefully considered. Ion-substituted systems, particularly those incorporating antimicrobial ions such as silver, copper, or zinc, may pose risks related to cytotoxicity or local ion overdose if release kinetics are not adequately controlled [45]. Excessive ion release has been associated with impaired cell viability and altered tissue responses, highlighting the need for precise compositional tuning and dosage control [46].

In addition, the long-term stability of doped and composite calcium phosphate systems remains a concern, as ion substitution and multiphase formulations may influence phase transformation behavior, degradation kinetics, and reproducibility during processing [46]. From a translational perspective, increased material complexity may also lead to higher production costs and regulatory challenges, particularly for systems incorporating bioactive additives or antimicrobial agents that require additional safety evaluation [47].

From a clinical evidence standpoint, most advanced calcium phosphate systems, including ion-substituted, amorphous, and composite formulations, are primarily supported by vitro and preclinical studies [48]. In contrast, conventional hydroxyapatite, β-tricalcium phosphate, and selected biphasic calcium phosphate materials benefit from more robust clinical documentation in dental regenerative applications [49,50]. As a result, while advanced material strategies demonstrate clear experimental potential, their routine clinical implementation remains limited by the scarcity of long-term, comparative clinical studies and standardized evaluation protocols.

To facilitate a concise overview of the diversity of advanced and modified calcium phosphate-based dental materials, their main categories, representative examples, and functional relevance are summarized in Table 2. This table highlights key material-level strategies employed to enhance biological performance, mechanical behavior, and applicability within digitally assisted dental workflows.

### 2.3. Digital Manufacturing Techniques for Calcium Phosphate-Based Dental Materials

By eliminating some of the drawbacks of more traditional methods of fabrication, digital manufacturing has greatly increased the processing options for dental materials based on calcium phosphate. Because of their inherent fragility and structure-dependent biological performance, geometric complexity, internal architectural control, and repeatability are crucial factors for calcium phosphate systems [51]. However, these aspects are generally limited by traditional manufacturing processes. Contrarily, with digitally assisted manufacturing, material distribution, scaffold geometry, and patient-specific design may be precisely controlled [52].

Because of its capacity to produce intricate, porous, and highly individualized structures, additive manufacturing has attracted a lot of interest among digital fabrication techniques for materials based on calcium phosphate [53].

Composite formulations and calcium phosphate pastes are ideal candidates for extrusion-based techniques like robocasting and direct ink writing because these methods enable the controlled deposition of material layers and the formation of linked pore networks [54]. There have been investigations into using binder jetting techniques on calcium phosphate powders; these methods have the potential to improve scalability and form complexity, but post-processing is still necessary to achieve adequate mechanical integrity [55].

Because of the critical roles played by porosity, interconnectivity, and anatomical conformity in biological integration, these additive techniques are of particular relevance in regenerative dental applications [56].

An alternate digital pathway for materials containing calcium phosphate is represented by subtractive manufacturing processes, which are mostly CAD/CAM milling-based [57]. If you need a very precise shape or a smooth finish on your prefabricated blocks or composite systems that include calcium phosphates, milling is the way to go. Ceramic calcium phosphate phases are naturally brittle, which limit tool wear, machining parameters, and possible geometries [58,59]. Subtractive methods are still best used in conjunction with other manufacturing procedures and have a narrower application to pure calcium phosphate ceramics [60].

One potential answer to the drawbacks of additive and subtractive manufacturing processes is the rise of hybrid systems that combine the two [61,62]. Hybrid workflows allow for better control over macro- and microstructural aspects by integrating the geometric freedom of additive manufacturing with the precision and surface refinement of subtractive methods [63]. For patient-specific applications involving calcium phosphate-based systems, these solutions are especially pertinent since they address anatomical precision, structural stability, and biological performance all at once [64,65].

From a translational standpoint, the implementation of digital manufacturing techniques for calcium phosphate-based dental materials is influenced by several practical constraints [66,67]. Additive and hybrid manufacturing workflows often involve higher initial costs related to specialized equipment, software infrastructure, and material preparation, which may limit accessibility in routine clinical settings [67]. In addition, post-processing requirements, including sintering and surface finishing, contribute to overall production costs and time [68].

Workflow reproducibility represents another critical consideration, as variations in feedstock formulation, printing parameters, and post-processing conditions can lead to inconsistencies in structural and mechanical outcomes [69]. Achieving reliable reproducibility requires standardized protocols, rigorous process control, and operator expertise [70].

Furthermore, the clinical adoption of digitally manufactured calcium phosphate systems is associated with a learning curve for both clinicians and dental technicians. Effective integration into clinical workflows depends on coordinated digital planning, material handling familiarity, and close collaboration between clinicians, engineers, and laboratory personnel [71]. These factors play a decisive role in determining whether digital manufacturing approaches can be translated from experimental settings into predictable clinical solutions [72,73].

The variability in digital manufacturing routes and their respective advantages and limitations for calcium phosphate-based dental materials is provided in Table 3:

### 2.4. Processing Strategies and Material Optimization

When it comes to the mechanical and biological performance of dental materials made of calcium phosphate, porosity is a crucial structural property. Promoting vascularization, cellular infiltration, and new bone formation, all while impacting mechanical stability and volume preservation, is crucial in regenerative dentistry, and porous structures play an important role in this process [74]. The overall osteoconductive behavior of calcium phosphate systems is influenced by both macro and microporosity. Macroporosity allows for tissue ingrowth, while microporosity improves surface area and ion exchange [75].

Digitally assisted manufacturing techniques enable a level of architectural control that is difficult to achieve using conventional fabrication methods. In particular, additive manufacturing approaches allow for predefined pore size, shape, and interconnectivity to be incorporated into scaffold designs, supporting more predictable biological integration [76]. This capability is especially relevant for dental applications such as alveolar ridge augmentation, sinus floor elevation, and periodontal defect reconstruction, where anatomical constraints and site-specific requirements necessitate controlled scaffold geometry [77].

On the other hand, mechanical strength naturally decreases as porosity increases, especially in systems of fragile ceramics like calcium phosphates. Therefore, finding the ideal balance between mechanical robustness and biological functionality is still very much a difficult task [78]. The rational design of dental calcium phosphate scaffolds relies on processing procedures that change pore architecture while keeping sufficient load-bearing capacity. The significance of optimizing digitally produced calcium phosphate systems through architecture is highlighted by these concerns [79].

From a practical and translational perspective, several quantitative benchmarks have been proposed in the literature to guide the design of digitally manufactured calcium phosphate scaffolds for dental applications [76]. Porosity levels in the range of approximately 50–80% are commonly reported as favorable for promoting vascularization and bone ingrowth, although increasing porosity is typically associated with reduced mechanical strength [74,75]. Macropore sizes between 200 and 600 μm are frequently considered optimal for bone tissue regeneration, while the presence of additional microporosity (<10 μm) may further enhance ion exchange and biological interactions [75].

In terms of mechanical performance, compressive strength values reported for porous calcium phosphate scaffolds intended for non-load-bearing dental applications generally range from 1 to 10 MPa, depending on porosity, phase composition, and processing conditions [80]. These values are substantially lower than those of dense ceramics, but are considered sufficient for space maintenance and regenerative procedures when combined with appropriate clinical stabilization. Such quantitative ranges provide practical guidance for balancing biological performance and structural integrity, while underscoring the need for application-specific optimization.

In addition to architectural design, phase stability and microstructural evolution represent key determinants of the long-term performance of calcium phosphate-based dental materials [80].

Processing conditions associated with digitally assisted fabrication, particularly post-fabrication thermal treatments, can induce phase transformations, grain growth, and changes in crystallinity. These microstructural modifications directly influence dissolution behavior, resorption kinetics, and mechanical reliability [81]. For example, highly crystalline hydroxyapatite phases tend to exhibit improved chemical stability, whereas less crystalline or multiphasic systems may demonstrate enhanced resorption and biological remodeling, which can be advantageous in regenerative dental applications [82].

The optimization of materials is enhanced by post-processing procedures, which stabilize the scaffold architecture and refine the surface features. Although microporosity and bioactivity can be compromised by over-densifying calcium phosphate ceramics, sintering is still necessary to achieve adequate densification and mechanical integrity [83,84]. Thus, structural resilience and preservation of physiologically relevant traits require delicate balancing acts in processing techniques. In addition to affecting early-stage cellular responses, surface-related changes made during or after processing may also affect wettability, roughness, and interfacial interactions with neighboring tissues [85].

The mechanical analysis of calcium phosphate scaffold load-bearing capability is governed by the interaction between microstructure, manufacturing history, and porosity. By distributing materials more logically throughout the scaffold design, digitally manufactured systems can reinforce stress-bearing areas while leaving porous sections unreinforced, facilitating biological integration [86]. Ceramic calcium phosphate phases are inherently fragile, which makes it extremely difficult to achieve consistent mechanical performance [87].

### 2.5. Clinical Relevance and Practical Considerations

While calcium phosphate-based materials and digitally assisted manufacturing strategies have demonstrated advantages at the material and structural levels, their translation into routine dental practice remains influenced by several practical and clinical considerations [88]. In dentistry, calcium phosphate materials are primarily employed in regenerative procedures, including alveolar ridge preservation, sinus floor elevation, periodontal regeneration, and peri-implant defect management.

A new bioceramic material showed somewhat better preservation of horizontal bone thickness compared to a bovine-derived xenograft material in the randomized study conducted by Wang et al. [89]. This was observed both immediately after the operation and 180 ± 14 days later, indicating the stability of the dimensional outcomes. The outcomes of injectable biphasic calcium phosphate (BCP) and anorganic bovine bone (ABB) were found to be similar by Tomas et al. [90]. The researchers found that BCP had a lower residual biomaterial percentage (28.61 ± 11.38%) compared to ABB (31.72 ± 15.52%), but these differences were not statistically significant (*p* > 0.05). As a post-extractive filler, nanocrystalline hydroxyapatite (Ostim^®^) was evaluated in the clinical experiment conducted by Canuto RA et al. [91]. Early epithelialization (day 7) was a hallmark of the healing process.

Greater clinical and radiographic improvement was demonstrated by Gupta et al. [92] when they expanded the assessment of hydroxyapatite/ſ-TCP to periodontal abnormalities compared to open flap debridement.

The combination of rhBMP-2/BioCaP/β-TCP resulted in a considerably larger new bone area (21.18% ± 7.62%) and a significantly less amount of leftover material (10.04% ± 4.57%) compared to β-TCP alone, as demonstrated by Sun et al. [93]. In these contexts, predictable biological behavior, handling characteristics, and volumetric stability are critical determinants of clinical success [94].

Although the reported clinical outcomes of calcium phosphate-based materials are generally encouraging, several limitations must be acknowledged when interpreting these findings. Many available clinical studies are characterized by relatively small sample sizes and short-to medium-term follow-up periods, which limit the robustness and generalizability of the reported outcomes [94,95]. In addition, heterogeneity in study design, defect morphology, surgical protocols, and outcome assessment methods introduces potential confounding factors that may influence clinical results [93,94,95,96].

Furthermore, a substantial proportion of the clinical evidence originates from non-randomized or single-center studies, which may be associated with selection bias and limited external validity. While these studies provide valuable preliminary insights, their findings should be interpreted with caution, underscoring the need for larger, well-designed randomized clinical trials with standardized protocols and extended follow-up to more reliably assess the clinical performance of calcium phosphate-based dental materials, particularly those manufactured using digital workflows.

Although digitally manufactured calcium phosphate systems show significant promise for improved anatomical fit and patient-specific design, they are still in the early stages of clinical acceptance due to issues such as high costs, complicated manufacturing processes, and the lack of standardized processing techniques [94]. Ceramic calcium phosphate phases are fragile and should only be used in very specific situations; they are not recommended for load-bearing indications that do not require extra mechanical support.

This means that a lot of calcium phosphate structures made digitally are still in the early stages of translational research or preclinical testing [95].

From a regulatory and practical standpoint, consistency in material properties, reproducibility of fabrication processes, and long-term stability must be demonstrated before widespread clinical implementation can be achieved. In addition, clinician familiarity with digitally manufactured biomaterials and their handling characteristics represents an important, yet often overlooked, aspect of translational success [96].

## 3. Future Perspectives and Limitations

The integration of digitally assisted production techniques with enhanced material design is anticipated to accelerate future advancements in dental materials based on calcium phosphate.

Beyond incremental improvements in fabrication hardware, future progress in digitally manufactured calcium phosphate-based dental materials is expected to increasingly rely on data-driven and computational approaches. Artificial intelligence-assisted design tools may enable the optimization of scaffold architecture, porosity gradients, and phase composition by integrating material properties, processing parameters, and biological performance into predictive design frameworks.

In parallel, the development of personalized biomaterial libraries—linking patient-specific anatomical, biological, and clinical data with predefined material formulations and digital manufacturing protocols—may support more individualized regenerative strategies. Such libraries could facilitate the selection of calcium phosphate compositions and architectures tailored to specific defect types and clinical indications.

Additionally, automated validation and quality control workflows, incorporating in-line monitoring, digital twins, and standardized performance metrics, may improve the reproducibility and regulatory compliance of digitally manufactured calcium phosphate systems. These advances have the potential to reduce variability, accelerate translational pathways, and support more predictable clinical implementation, provided that they are coupled with rigorous experimental and clinical validation.

For better mechanical stability, resorption behavior, and biological responsiveness, one important aim is to improve compositionally tailored calcium phosphate systems, which can include ion-substituted, multiphasic, and composite formulations. To optimize these systems for specific dental indications, a better understanding of structure–property correlations is necessary.

The manufacturing industry may look forward to better control over scaffold architecture, porosity gradients, and patient-specific geometries thanks to ongoing advancements in additive and hybrid fabrication processes. To improve repeatability and decrease variability associated with current experimental methodologies, more predictable fabrication workflows may be enabled by integrating digital design tools with material-level optimization. At the same time, it is anticipated that attempts to standardize processing parameters and post-processing techniques would be crucial in enabling more widespread clinical usage.

Future progress in this field will depend on interdisciplinary collaboration between materials scientists, engineers, and dental clinicians. By aligning material innovation with clinically relevant requirements and standardized manufacturing approaches, digitally fabricated calcium phosphate-based dental materials may achieve more reliable and predictable translation into routine dental practice.

This narrative review has several limitations that should be acknowledged. First, the analysis is based on a qualitative synthesis of the published literature rather than a systematic review approach. As such, article selection was guided by relevance and scientific contribution, which may introduce an element of selection bias and does not allow for quantitative comparison of outcomes across studies.

Although calcium phosphate-based materials are widely used in dentistry, the body of evidence addressing advanced material systems and digitally manufactured calcium phosphate constructs remains heterogeneous. Differences in material composition, fabrication techniques, processing parameters, and evaluation protocols limit direct comparison between studies and preclude definitive conclusions regarding optimal material configurations.

A substantial proportion of the available evidence for digitally manufactured calcium phosphate materials originates from in vitro and preclinical investigations. Clinical data, particularly long-term and comparative studies, remain limited, restricting the ability to draw firm conclusions regarding clinical effectiveness and standardized indications.

Finally, variations in reporting standards and outcome measures across studies may affect the generalizability of the findings. These limitations highlight the need for standardized fabrication protocols, harmonized evaluation methodologies, and well-designed clinical studies to support the robust translation of advanced calcium phosphate-based materials into routine dental practice.

## 4. Conclusions

The inherent bioactivity, compositional diversity, and established usage of calcium phosphate-based materials in regeneration operations ensure that they continue to be a cornerstone of dental biomaterials.

In order to overcome constraints in mechanical performance, resorption behavior, and biological responsiveness, various novel material systems have been developed, going beyond traditional stoichiometric phases. These systems include ion-substituted, composite, and amorphous or metastable calcium phosphates. These changes at the material level greatly increase the variety of functions that calcium phosphate systems can perform that are useful in dentistry.

Clinical studies confirm the applicability of conventional calcium phosphate materials in dental regeneration, while evidence supporting digitally manufactured and advanced systems remains heterogeneous and largely non-comparative. Overall, current data indicate that further material optimization and well-designed clinical studies are required before digitally fabricated calcium phosphate-based materials can be considered a reliable alternative to established regenerative solutions in routine dental practice.

From a practical perspective, several take-home messages can be derived from the present review. First, the clinical use of calcium phosphate-based materials should be guided by phase-dependent behavior, with material selection tailored to the required balance between resorption rate and structural stability. Second, digitally assisted manufacturing offers clear advantages in terms of patient-specific design and architectural control, but its successful clinical translation depends on standardized processing protocols and adequate post-processing to ensure reproducible performance. Finally, while advanced and digitally manufactured calcium phosphate systems show significant experimental promise, their routine clinical adoption requires further well-designed clinical studies providing long-term and comparative evidence.

## Figures and Tables

**Table 1:** Summary of key findings related to digitally manufactured calcium phosphate-based materials used in dental applications.

Calcium Phosphate Phase	Resorbtion Behavior	Mechanical Stability	Clinical Situations Where It Performs Best	Main Clinical Limitations
Hydroxyapatite (HA)	Very low resorption; long-term persistence	High chemical stability; moderate mechanical strength	Space maintenance in regenerative procedures; volume preservation; coating or filler where long-term stability is required [18]	Limited or absent resorption; residual material may persist long-term; limited remodeling [19].
α-Tricalcium phosphate (α-TCP)	Rapid resorption; hydrolyzes to apatite in vivo	Low intrinsic mechanical stability	Early stage defect filling; applications requiring rapid bone replacement and remodeling [12].	Limited volumetric stability; not suitable where prolonged mechanical support is needed [15].
β-Tricalcium phosphate (β-TCP)	Moderate resorption; gradual degradation	Lower mechanical stability than HA	Regenerative procedures requiring progressive resorption and sustained osteoconductive support [13].	Insufficient mechanical resistance in load-exposed sites.
Biphasic calcium phosphate (HA/β-TCP)	Tunable resorption depending on phase ratio	Intermediate stability	Indication-driven applications balancing stability and resorption [13,14].	Requires careful phase ratio optimization; heterogeneous clinical outcomes.

**Table 2:** Categories, Structural Characteristics, and Functional Outcomes of Modified Calcium Phosphate–Based Materials in Dentistry.

Material Category	Representative Examples	Processing- or Structure-Related Findings	Functional Outcomes Relevant to Dentistry	Key Limitations
Ion-substituted calcium phosphates	Sr-, Mg-, Zn-, Si-, Ag-doped CaP	Modified crystallinity and solubility influencing resorption kinetics and surface reactivity	Enhanced osteogenic response; potential antibacterial effects; improved biological integration [45]	Limited clinical validation; compositional heterogeneity
Composite calcium phosphate systems	CaP–polymer composites (PLA, PCL, biopolymers); CaP–bioglass systems	Improved mechanical stability and processability facilitating digitally assisted fabrication	Suitable for regenerative applications and patient-specific constructs [46,47]	Complex fabrication protocols; variable degradation behavior
Amorphous and metastable calcium phosphate phases	Amorphous calcium phosphate (ACP); metastable CaP phases	High solubility and rapid ion release affecting early mineralization processes	Remineralization; early-stage mineral deposition; apatite precursor role [48]	Limited structural stability; short-term functionality
Functional forms and material configurations	Granules; cements; pastes; injectable formulations; printable inks and slurries	Application-specific handling and compatibility with digital workflows	Broad adaptability to conventional and digitally assisted dental applications [49,50]	Limited standardization across formulations

**Table 3:** Manufacturing Techniques for Calcium Phosphate–Based Dental Materials: Processing Characteristics, Clinical Applications, and Limitations.

Manufacturing Technique	Material Form	Key Processing-Related Findings	Functional Relevance in Dentistry	Main Limitations
Extrusion-based printing / Robocasting	Calcium phosphate pastes, inks, composites	Ink rheology and extrusion parameters enable controlled pore architecture and shape fidelity [66].	Patient-specific scaffolds for bone regeneration and defect reconstruction.	Requires optimized formulations; post-processing critical for mechanical stability [67].
Binder jetting	Calcium phosphate powders	High geometric complexity is achievable; part properties strongly dependent on post-print sintering [68].	Custom bone grafts and complex geometries for regenerative applications.	Low green strength: extensive post-processing required [69].
Subtractive manufacturing (CAD/CAM milling)	Prefabricated CaP-containing blocks or composites	High dimensional accuracy and surface quality; limited internal architecture [70].	Custom-fit bone blocks and prosthetic components.	Material waste; fracture risk due to ceramic brittleness [71].
Hybrid manufacturing approaches	Additively fabricated CaP structures with subtractive finishing	Combination of geometric freedom and surface refinement [72].	Improved fit and surface precision in patient-specific constructs.	Increased process complexity; limited clinical standardization [73].

## Data Availability

No new data were created or analyzed in this study. Data sharing is not applicable to this article.

## Abbreviations

The following abbreviations are used in this manuscript:

HA	hydroxyapatite
TCP	Tricalcium phosphate
α-TCP	α-Tricalcium phosphate
CDHA	Decomposition of Carbonated Apatite
BCP	Biphasic Calcium Phosphate
ABB	Anorganic Bovine Bone
rhBMP	recombinant human Bone Morphogenetic Protein
